# Coenzyme Q10 supplementation in burn patients: a double-blind placebo-controlled randomized clinical trial

**DOI:** 10.1186/s13063-024-08006-y

**Published:** 2024-03-02

**Authors:** Zahra Kiani, Nadereh Khorsand, Fahimeh Beigi, Gholamreza Askari, Manoj Sharma, Mohammad Bagherniya

**Affiliations:** 1 Nutrition and Food Security Research Center and Department of Community Nutrition, School of Nutrition and Food Science, Isfahan University of Medical Sciences, Isfahan, Iran; 2 Department of Internal Medicine, Imam Musa Kazem Hospital, Isfahan University of Medical Sciences, Isfahan, Iran; 3 Pharmaceutical Biotechnology Department, School of Pharmacy and Pharmaceutical Sciences, Isfahan University of Medical Science, Isfahan, Iran; 4 Research and Development Unit, Imam Muss Kazim Hospital, Isfahan University of Medical Science, Isfahan, Iran; 5 Anesthesia and Critical Care Research Center, Isfahan University of Medical Sciences, Isfahan, Iran; 6 Department of Social and Behavioral Health, School of Public Health, University of Nevada, Las Vegas, Las Vegas, NV USA

**Keywords:** Coenzyme Q10, Burn, Inflammation, Oxidative stress

## Abstract

**Background:**

Burn injuries are important medical problems that, aside from skin damage, cause a systemic response including inflammation, oxidative stress, endocrine disorders, immune response, and hypermetabolic and catabolic responses which affect all the organs in the body. The aim of this study was to determine the effect of coenzyme Q10 (CoQ10) supplementation on inflammation, oxidative stress, and clinical outcomes in burn patients.

**Methods:**

In a double-blind placebo-controlled randomized clinical trial, 60 burn patients were randomly assigned to receive 100 mg CoQ10 three times a day (total 300 mg/day) or a placebo for 10 days. Inflammatory markers including erythrocyte sedimentation rate (ESR), C-reactive protein (CRP), oxidative stress markers including total antioxidant capacity (TAC), malondialdehyde (MDA) and superoxide dismutase (SOD) activity, fasting blood glucose (FBG), blood urea nitrogen (BUN), creatinine, white blood cells (WBC), and body temperature were assessed as primary outcomes and albumin, prothrombin time (PT), partial thromboplastin time (PTT), international normalized ratio (INR), other hematological parameters, blood pressure, O_2_ saturation, ICU duration, and 28-mortality rate were assessed as secondary outcomes.

**Results:**

Fifty-two participants completed the trial. CRP and ESR levels were not significantly different between CoQ10 and placebo groups at the end of the study (*P* = 0.550 and *P* = 0.306, respectively). No significant differences between groups were observed for TAC (*P* = 0.865), MDA (*P* = 0.692), and SOD activity (*P* = 0.633) as well. Administration of CoQ10 resulted in a significant increase in albumin levels compared to placebo (*P* = 0.031). There was no statistically significant difference between the two groups in other measured outcomes (*P* > 0.05).

**Conclusion:**

Results showed that in patients with burn injury, CoQ10 administration had no effect on inflammatory markers and oxidative stress, although serum albumin levels were improved after supplementation. Further studies with albumin as the primary outcome are needed to confirm this finding.

## Introduction

Around the world, many people are hospitalized due to burns every year. After traffic accidents, falls, and interpersonal violence, burns are the fourth most common type of trauma [1]. Burns are types of injuries that are categorized as thermal, electrical, chemical, and radiation burns based on causative agents [2]. These not only have physical, psychological, emotional, and spiritual adverse consequences for the patients and their families but also impose a heavy economic burden on the healthcare system [2, 3]. These are one of the main causes of morbidity and mortality especially in low and middle-income countries [4]. According to the result of a recent epidemiological study, in 2019, 111,292 deaths had been reported globally, which were related to burns [3]. However, epidemiological characteristics of burn injuries are generally different among continents, which is possibly due to different infrastructure and circumstances in the continents [4]. Burn injury is a complex medical problem. Coagulative necrosis occurs because of burn injury in different layers of skin and other tissues. Several factors like temperature, transmitted energy, and duration of exposure can affect the depth of damage [5]. Aside from skin damage, severe burn injury causes a systemic response that affects all the organs in the body [6]. Burn’s pathophysiology involves severe inflammation, oxidative stress, endocrine disorders, immune response, and hypermetabolic and catabolic responses [7, 8]. To enhance the process of healing, immediately after burn injury, an inflammatory response is initiated, and the levels of inflammatory mediators increase in the body. However, if this inflammation response becomes severe and uncontrolled, it does not contribute to healing, but rather the excessive release of cytokines and other inflammatory mediators causes systemic inflammatory response syndrome (SIRS) which can lead to catabolism, organ failure, infection, and even death [9]. The concentration of inflammatory mediators such as IL-8, IL-6, and CRP can remain high for long periods after burn injury [10]. High levels of oxidative stress following burn damage have been observed in both animals and humans [11]. It has been shown that free radicals have positive effects on antimicrobial activities and wound healing. However, following burn damage, an imbalance between oxidant generation and antioxidant mechanisms occurs due to a lack of antioxidants or excessive production of oxidants. This can lead to inflammation and other harmful outcomes [6, 12]. Therefore, a burn patient may suffer from various fatal complications such as burn shock, sepsis, infection, imbalance of electrolytes, multiple organ failure, immune dysfunction, muscle wasting, and cachexia [6, 13–15]. Given these points, burn management has a lot of complexities; thus, it needs a team approach. Nutritional care is of special importance in burn management. Inflammation and oxidative stress caused by burn injury result in the depletion of the endogenous antioxidant defense system [7, 8]. Studies have shown that supplementation with several micronutrients has beneficial effects on inflammatory response and antioxidant status and can improve clinical outcomes and wound healing [16, 17]. Researchers are trying to discover the antioxidant properties of new compounds and use them for this purpose [18, 19].

Coenzyme Q10 (CoQ10) is a lipophilic vitamin-like compound consisting of a benzoquinone ring and a side chain of 10 isoprene units [20, 21]. CoQ10 is endogenously synthesized from tyrosine and it has fundamental functions in the body. It has a role in metabolic processes, ATP generation, electron transportation in the mitochondrial electron transport chain, protection of cells from oxidative damage as a potent antioxidant, and regulation of the expression of genes related to inflammation [22]. It is stated that after fish oil and multivitamin, CoQ10 is the most widely used nutrition supplement [20]. The efficacy of CoQ10 supplementation in various diseases has been investigated before [23–29]. Decreased levels of CoQ10 have been observed in sepsis and critically ill patients [30–32]. In an animal study, it was shown that mitochondrial dysfunction, oxidative stress, metabolic dysfunction, inflammation, and insulin resistance were improved as a result of CoQ10 intake in burned mice [33].

Based on this evidence, it seems that CoQ10 supplementation may be effective in alleviating inflammation and oxidative stress and improving clinical outcomes in burn patients. Thus, we performed a randomized double-blind placebo-controlled trial to investigate the effect of CoQ10 supplementation for 10 days on inflammatory and oxidative stress markers and clinical outcomes of burn patients admitted to the intensive care unit (ICU).

## Methods

### Study design

This study was a parallel randomized, double-blind, placebo-controlled trial assessing the effect of CoQ10 supplementation in burn patients admitted to the ICU compared to the placebo. The study was conducted in Imam Musa Kazem Hospital, the burn center in Isfahan, Iran, from June 2021 to March 2022. The study protocol was approved by the ethics committee of the Isfahan University of Medical Sciences (IR.MUI.RESEARCH.REC.1400.109). Our clinical trial was registered at IRCT.ir (IRCT20201129049534N3). Patients or next of kin provided written informed consent for participating in this study.

### Study population

Patients admitted to the ICU of the center were included in the study if they had the following criteria: (1) 18 to 65 years old, (2) with 20 to 60% of total body surface area (TBSA) burn, and (3) had gastrointestinal tract with normal function. Subjects were excluded if they had the following criteria: (1) pregnancy, (2) severe sepsis or septic shock, (3) hypovolemic shock, (4) prediction of death in the first week after admission, (5) immunodeficiency disease, liver cirrhosis, or pancreatitis, (6) and not providing consent. There were no limitations for the degree and cause of burns. The evaluation of eligibility and enrollment of patients was done by two researchers (Z.K and N.Kh).

### Randomization and blinding

Eligible subjects were randomly allocated to the intervention or control group in a ratio of 1:1 with a block size of four based on age and gender. Allocation sequences were determined by an independent statistician using a random number table. Then, they were kept in opaque, sealed, and numbered envelopes until the end of eligibility criteria evaluation. Treatment assignments were concealed from patients and investigators until data analysis was completed. CoQ10 and placebo capsules were packed in similar boxes and labeled as A and B by the pharmaceutical company. The capsules were completely identical in terms of appearance properties including color, size, shape, and odor. Patients, researchers, physicians, nurses, laboratory staff, and data analysts were blinded until data analysis was completed at the end of the study.

### Supplement dosage

Different doses of CoQ10 can be used depending on the indication. However, in some medical conditions, it is usually between 60 to 1200 mg per day. It has been shown that CoQ10 application is safe and tolerable up to a dose of 1200 mg per day [20]. Supplement dosage was determined based on a meta-analysis study, which has shown that CoQ10 supplementation lowers inflammatory factor levels, particularly at high dosages (higher or equal to 200 mg/day). Due to insufficient data on burn patients, 300 mg per day was chosen to avoid possible adverse effects [34].

### Intervention

Eligible burn patients were included in this study after 24 to 48 h of hospitalization in the ICU with stable hemodynamic status. Patients were randomly assigned to receive 100 mg CoQ10 three times a day (total 300 mg/day) (Dana pharmaceutical company, Tabriz, Iran) or placebo (maltodextrin) for the same dose, after meals for 10 days. All the participants had oral nutrition during the study period, and their energy intake was the same, about 35–40 kcal/kg. Patients in both groups were visited daily by a physician, and possible adverse effects (including gastrointestinal symptoms or any other adverse event attributed to intervention) were evaluated and reported by the physician. Participants received standard burn treatments and medications as prescribed by their physician. We had no intervention in this regard. Patients were followed up in person by one of the researchers (Z.K) in terms of receiving all the doses of CoQ10 and placebo.

### Sample size

Considering CRP as a main outcome and according to a prior study [35], considering the type I error of 5% (*α* = 0.05) and the type II error of 20% (*β* = 0.20) with a test power of 80%, and standardized effect size of 15 (*Δ* = 15), the sample size was calculated as 30 patients in each group, and a total of 60 patients were included.

$$n= \frac{2\left[{\left({Z}_{1-\frac{\alpha }{2}}+{Z}_{1-\beta }\right)}^{2}\times {S}^{2}\right]}{{\Delta }^{2}} = \frac{2\left[{\left(1.96+0.84\right)}^{2}\times {\left(21\right)}^{2}\right]}{{15}^{2}}=30$$

### Assessment

Blood samples were collected from all participants at baseline and at the end of the study. Samples were centrifuged at 3600 rpm for 3 to 4 min, and the separated serum was stored at − 80 °C until analysis. Inflammatory markers including erythrocyte sedimentation rate (ESR), C-reactive protein (CRP), oxidative stress markers including total antioxidant capacity (TAC), malondialdehyde (MDA) and superoxide dismutase (SOD) activity, fasting blood glucose (FBG), blood urea nitrogen (BUN), creatinine, white blood cells (WBC), and body temperature were assessed as primary outcomes, and albumin, prothrombin time (PT), partial thromboplastin time (PTT), international normalized ratio (INR), other hematological parameters, blood pressure, O_2_ saturation, ICU duration, and 28-mortality rate were assessed as secondary outcomes. Levels of BUN, creatinine, FBG, albumin, and CRP were measured with enzyme-linked immunosorbent assay (ELISA) method. Albumin was assessed by immunochemical method. Oxidative stress factors including TAC, MDA, and SOD activity were measured by calorimetric method using commercial kits (Kiazist, Iran). ESR was assessed by *Westergren method*, and hematological parameters including red blood cells (RBC), hemoglobin, hematocrit, mean corpuscular volume (MCV), mean corpuscular hemoglobin (MCH), mean corpuscular hemoglobin concentration (MCHC), WBC, lymphocyte, neutrophil, and platelet levels were measured at the Clinical Chemistry Laboratory in Imam Musa Kazem Hospital, by an automated hematology analyzer. Blood pressure, body temperature, and O_2_ saturation were measured and recorded by a nurse every 2 h every day. Medications and nutritional supplement intake, demographic, anthropometric, and other required clinical data were collected from patients’ medical records in the hospital. Length of a hospital stay and 28-day mortality rate were recorded as well.

### Statistical analysis

Statistical analyses were performed with the SPSS software (version 23). The normality of the variables’ distribution was assessed using the Kolmogorov-Simonov test and the Skewness index. Continuous variables were reported as means with SD (standard deviation). Frequency and percentage were reported for categorical variables. Within-group changes (baseline versus post-intervention) were evaluated using paired *t*-tests. Analysis of covariance (ANCOVA) was used to evaluate between-group differences (adjusted for baseline values). *P*-value < 0.05 was considered to be statistically significant.

## Results

### Study population

**Fig. 1:**
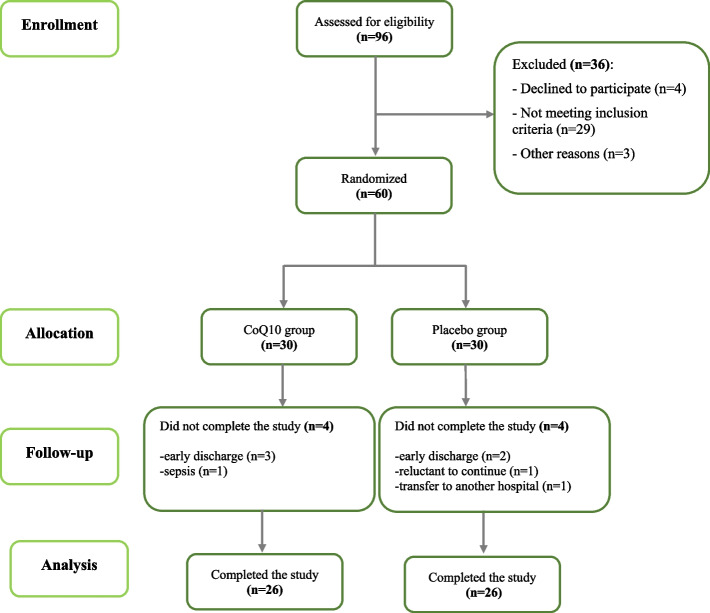
CONSORT flow diagram of the study

From the initially enrolled burned patients, 60 patients entered the clinical trial and were randomized into CoQ10 (*n* = 30) or placebo (*n* = 30) groups. The baseline characteristics of these 60 patients are presented in Table 1. Four subjects in the CoQ10 group (3 subjects due to early discharge and 1 subject due to sepsis) and 4 subjects in the placebo group (2 subjects due to early discharge, 1 subject due to reluctant to continue the study, and 1 subject due to transfer to another hospital) did not complete the study. Finally, 52 patients (26 in each group) were included in the analysis (Fig. 1). No serious side effect related to the intervention was reported in either group. There was no difference between the two study groups in terms of baseline characteristics as shown in Table 1. The mean age of participants was 34.90 ± 10.77and 36.77 ± 11.00 in CoQ10 and placebo groups, respectively (*P* = 0.509). The average TBSA of included patients was 48.17 ± 9.87 and 44.27 ± 10.45 in CoQ10 and placebo groups, respectively (*P* = 0.143). In the CoQ10 group, 30% of participants were female, and in the placebo group, 30% of participants were also female. Most burns were caused by flame, 93.3% in the CoQ10 group and 76.7% in the placebo group. All the patients received their routine care including nutritional supplementation. CoQ10 and placebo groups did not significantly differ in terms of nutritional supplements, anti-inflammatory drugs, and statins intake (Table 1).

**Table 1:** Baseline characteristics of the study population

			**CoQ10 (*n* = 30)**	**Placebo (*n* = 30)**	***P*-value**
Age (years)			34.90 ± 10.77	36.77 ± 11.00	0.509^a^
Sex *n* (%)	Female		9 (30%)	9 (30%)	0.99^b^
	Male		21(70%)	21(70%)	
Weight (kg)			74.43 ± 14.98	71.67 ± 11.58	0.427^a^
Height (cm)			168.93 ± 9.23	171.17 ± 7.62	0.311^a^
BMI (kg/m^2^)			26.23 ± 5.57	24.43 ± 3.51	0.141^a^
TBSA (%)			48.17 ± 9.87	44.27 ± 10.45	0.143^a^
Burn degree *n* (%)	2nd degree		7 (23.3%)	7 (23.3%)	0.641
	3rd degree		6 (20%)	9 (30%)	
	2nd and 3rd degree		17 (56.7%)	14 46.7(%)	
Cause *n* (%)	Flame		28 (93.3%)	23 (76.7%)	0.145^b^
	Others^c^		2 (6.7%)	7 (23.3%)	
Comorbidities *n* (%)	No		24 (80%)	25 (83.3%)	0.99^b^
	Yes	Total	6 (20%)	5 (16.7%)	
		Hypertension	4 (13.3%)	0	
		Hyperlipidemia	0	1 (3.3%)	
		Hypertension-kidney disease	0	1 (3.3%)	
		Lung disease	1 (3.3%)	2 (6.7%)	
		Hypothyroidism	1 (3.3%)	0	
		Diabetes	0	1 (3.3%)	
Protein intake (g/d)			142.27 ± 21.68	136.47 ± 16.90	0.253^a^
Energy intake (kcal/day)			3170.00 ± 557.18	2934.50 ± 555.27	0.106^a^
Supplement intake *n* (%)	Vitamin C		30 (100%)	30 (100%)	-
	Vitamin A		27 (90%)	26 (86.7%)	0.99^b^
	Vitamin D		30 (100%)	30 (100%)	-
	Vitamin E		27 (90%)	26 (86.7%)	0.99^b^
	Vitamin B6		8 (26.7%)	5 (16.7%)	0.532^b^
	Vitamin B1		8 (26.7%)	13 (43.3%)	0.279^b^
	B-complex^d^		30 (100%)	30 (100%)	-
	Selenium		26 (86.7%)	22 (73.3%)	0.333^b^
	Zinc		30 (100%)	28 (93.3%)	0.492^b^
	Omega-3		15 (50%)	12 (40%)	0.604^b^
	Ferfolic^e^		17 (56.7%)	23 (76.7%)	0.170^b^
	Heallagen^f^		17 (56.7%)	14 (46.7%)	0.606^b^
	Albumin		20 (66.7%)	23 (76.7%)	0.567^b^
Drug intake *n* (%)	Corticosteroids		16 (53.3%)	17 (56.7%)	0.99^b^
	NSAIDs		15 (50%)	17 (56.7%)	0.796^b^
	Statins		2 (6.7%)	1 (3.3%)	0.99^b^

Data are shown as mean ± standard deviation or *n* (%)

*Abbreviations*: *BMI* body mass index, *TBSA* total body surface area, *NSAIDs*, non-steroidal anti-inflammatory drugs

^a^Comparison of Q10 and placebo group based on independent *t*-tests

^b^Comparison of Q10 and placebo group based on Fisher’s exact test

^c^Scald, chemicals, electricity

^d^Containing group B vitamins

^e^Containing iron and folic acid

^f^Containing L-arginine, L-glutamine, and calcium beta-hydroxy beta-methyl butyrate (HMB)

### Inflammatory markers

The differences in the CRP and ESR levels were not significant between CoQ10 and placebo groups (*P* = 0.550 and *P* = 0.306, respectively) (Table 2).

**Table 2:** Effects of coenzyme Q10 supplementation on study outcomes

	**Q10 (*n* = 26)**			**Placebo (*n* = 26)**			***P*-value (Between-group)**
	**Baseline**	**End**	***P*-value (within group)**	**Baseline**	**End**	***P*-value (within-group)**	
MDA (nmol/ml)	26.50 ± 3.98	26.92 ± 2.70	0.573	31.93 ± 7.02	31.03 ± 6.49	0.178	0.692
SOD activity (U SOD activity/ml)	14.33 ± 3.03	13.38 ± 2.87	0.197	13.40 ± 2.54	13.48 ± 4.66	0.715	0.633
TAC (nmol of trolox equivalent/ml)	32.25 ± 4.12	34.16 ± 10	0.412	32.45 ± 4.33	34.54 ± 7.90	0.247	0.865
CRP (mg/l)	128.21 ± 69.61	78.60 ± 63.72	0.002	133.15 ± 82.50	90.49 ± 68.57	0.01	0.550
ESR (mm)	57.27 ± 33.00	69.62 ± 22.54	0.098	50.27 ± 27.55	61.96 ± 22.86	0.059	0.306
Albumin (mg/dl)	2.93 ± 0.32	3.26 ± 0.48	0.008	2.86 ± 0.43	2.97 ± 0.42	0.180	**0.031**
PT	14.09 ± 1.30	14.10 ± 1.79	0.968	13.53 ± 0.92	14.16 ± 1.56	0.085	0.677
PTT	37.12 ± 17.42	36.69 ± 5.06	0.551	35.12 ± 5.05	39.92 ± 10.10	0.012	0.144
INR	1.14 ± 0.17	1.14 ± 0.24	0.99	1.07 ± 0.12	1.14 ± 0.17	0.102	0.795
BUN (mg/dl)	13.55 ± 2.74	13.44 ± 3.56	0.847	15.83 ± 6.18	15.20 ± 7.72	0.697	0.655
Cr (mg/dl)	0.87 ± 0.13	0.87 ± 0.14	0.879	0.91 ± 0.14	0.91 ± 0.14	0.99	0.575
FBG (mg/dl)	120.92 ± 66.17	93.12 ± 17.30	0.046	99.12 ± 27.45	94.00 ± 18.77	0.383	0.743
RBC (10^6^/µL)	3.90 ± 0.80	3.59 ± 0.55	0.020	3.75 ± 0.79	3.43 ± 0.40	0.021	0.346
Hemoglobin (g/dl)	11.12 ± 2.48	10.11 ± 1.47	0.021	11.17 ± 2.69	9.91 ± 1.28	0.010	0.524
Hematocrit (%)	33.55 ± 6.16	30.72 ± 3.74	0.016	33.72 ± 7.23	30.30 ± 3.43	0.009	0.598
MCV (fL)	85.94 ± 6.99	86.10 ± 5.30	0.754	88.28 ± 3.76	88.39 ± 3.68	0.789	0.304
MCH (pg)	28.39 ± 2.83	28.27 ± 2.02	0.618	29.10 ± 1.52	28.92 ± 1.49	0.439	0.514
MCHC (g/dl)	33.03 ± 1.94	32.56 ± 2.04	0.399	32.97 ± 1.36	32.72 ± 1.63	0.431	0.732
WBC (µL)	9519.23 ± 3604.78	9000 ± 2915.61	0.531	12161.54 ± 4883.94	10,396.15 ± 7255.59	0.275	0.605
Lymphocyte (%)	17.37 ± 7.87	19.62 ± 7.87	0.302	15.94 ± 7.19	16.75 ± 6.92	0.653	0.197
Neutrophil (%)	74.02 ± 8.69	72.75 ± 8.14	0.561	77.75 ± 10.53	76.75 ± 9.58	0.716	0.154
Platelet (10^3^/µL)	270.08 ± 138.23	409.19 ± 144.05	0.000	233.96 ± 95.96	376.08 ± 171.44	0.000	0.678
Mean temperature (°C)	37.44 ± 0.28	37.39 ± 0.34	0.488	37.36 ± 0.31	37.49 ± 0.41	0.118	0.172
Mean systolic BP (mmHg)	129.54 ± 10.93	124.77 ± 9.02	0.029	128.82 ± 12.57	125.43 ± 9.35	0.158	0.693
Mean diastolic BP (mmHg)	68.76 ± 8	70.55 ± 6.60	0.309	70.54 ± 8.06	68.44 ± 6.39	0.216	0.148
Mean O_2_ Sat (%)	95.83 ± 1.41	96.05 ± 1.26	0.567	95.79 ± 1.11	96.22 ± 1.17	0.165	0.610
ICU duration (days)	18.83 ± 10.72			23.60 ± 10.03			0.122^a^
28-day mortality (*N*)	2 (7.7%)			3 (11.5%)			0.99^b^

Data are shown as mean ± standard deviation or *n* (%)

Between-group and within-group differences were tested with ANCOVA and paired *t*-test respectively

*Abbreviations*: *MDA* malondialdehyde, *SOD* superoxide dismutase, *TAC* total antioxidant capacity, *CRP* C-reactive protein, *ESR* erythrocyte sedimentation rate, *PT* prothrombin time, *PTT* partial thromboplastin time, *INR* international normalized ratio, *BUN* blood urea nitrogen, *Cr* creatinine, *FBG* fasting blood glucose, *RBC* red blood cell, *MCV* mean corpuscular volume, *MCH* mean corpuscular hemoglobin, *MCHC* mean corpuscular hemoglobin concentration, *WBC* white blood cell, *BP* blood pressure, *O*_*2*_* Sat* oxygen saturation, *ICU* intensive care unit

^a^Comparison of Q10 and placebo group based on independent *t*-tests

^b^Comparison of Q10 and placebo group based on Fisher’s exact test

### Oxidative stress markers

MDA, SOD activity, and TAC were assessed as oxidative stress markers in the included patients. As shown in Table 2, the between-group comparison revealed no significant difference between CoQ10 and placebo groups at the end of the study for MDA (*P* = 0.692), SOD activity (*P* = 0.633), and TAC (*P* = 0.865).

### Hematological and biochemical parameters

Changes in hematological and biochemical parameters are reported in Table 2. Albumin concentration was higher in the subjects receiving CoQ10 compared to the placebo group (*P* = 0.031) at the end of the intervention.

No significant differences between groups were observed for BUN, creatinine, and FBG concentrations after intervention (*P* > 0.05).

As shown in Table 2, there were no significant differences in hematological parameters (RBC, hemoglobin, hematocrit, MCV, MCH, MCHC, WBC, lymphocyte, neutrophil, and platelet) between CoQ10 and placebo groups at the end of the study (*P* > 0.05). Likewise, no significant changes were observed regarding PT, PTT, and INR values in the CoQ10 group compared to the placebo group (*P* > 0.05).

### Other clinical outcomes

As reported in Table 2, no between-group differences were observed for systolic and diastolic blood pressure, body temperature, and O_2_ saturation (*P* > 0.05).

ICU length of stay in the CoQ10 group (18.83 ± 10.72) was shorter than the placebo group (23.60 ± 10.03); however, the difference was not statistically significant (*P* = 0.122). Moreover, no significant difference was observed in the 28-day mortality rate between the two groups (2 in the CoQ10 group vs. 3 in the placebo group) (*P* = 0.99). (Table 2).

## Discussion

Burn injury is a condition with complex pathophysiological alternations, both local and distant. Nutritional supplementation has always been considered one of the effective strategies in burn management. To the best of our knowledge, the current clinical trial is the first study evaluating the effect of CoQ10 supplementation in burn patients in Iran. The findings of the current study demonstrated that supplementation with 300 mg/day CoQ10 in burn patients for 10 days had no significant effect on our primary outcomes, but it could significantly improve the levels of albumin. The changes were not significant for other factors.

We assessed the effects of CoQ10 supplementation on MDA, SOD activity, and TAC as oxidative stress markers and ESR and CRP as inflammatory factors. CoQ10 is known as an antioxidant and free radical scavenger [21]. It is able to reduce and neutralize free radicals and ROS and is also involved in improving electron transport chain efficiency and vitamin E and C regenerating [36]. In addition, CoQ10 increases the activity of antioxidant enzymes including SOD, glutathione peroxidase (GPx), and catalase (CAT) by absorbing free radicals and increasing gene expression of the antioxidant enzymes [37]. Moreover, anti-inflammatory properties have been suggested for CoQ10. The main proposed mechanism of anti-inflammatory effects of CoQ10 is that it decreases nuclear factor kappa B (NF-kB)-dependent gene expression. Produced ROS can activate NF-kB which upregulates pro-inflammatory cytokines. Therefore, CoQ10 can decrease the expression of the pro-inflammatory cytokines by reducing free radicals [38]. Previously, in interventional studies, it has been shown that CoQ10 intake had beneficial effects in attenuating oxidative stress and inflammation [28, 37–40]. However, in our work, MDA, SOD activity, TAC, and ESR and CRP levels did not change significantly after CoQ10 supplementation.

The results of some prior studies are consistent with our findings. In a recent randomized, double-blind, placebo-controlled trial, Kuriyama et al. investigated the effect of 1800 mg ubiquinol-10 for 4 weeks in burn patients. They indicated that although intracellular CoQ10 content in peripheral blood mononuclear cells (PBMCs) and plasma concentrations of CoQ10 were raised, plasma levels of inflammatory markers did not significantly change because of CoQ10 supplementation [41]. In another study, administration of 200 mg ubiquinol twice a day for up to 7 days in patients with severe sepsis or septic shock could not reduce inflammatory markers [42]. In a clinical trial on NAFLD patients, TAC concentrations were reduced after 100 mg/day CoQ10 intake in 4 weeks. Furthermore, changes in MDA levels were not significant [43]. In a study by Gokbel et al., oral CoQ10 intake in a dose of 200 mg/day had no significant impact on MDA, oxidized LDL, SOD, and GPx in hemodialysis patients [44]. Results of another study by Okudan et al. also revealed no beneficial effects of 4-week supplementation with 200 mg/day CoQ10 on SOD activity and MDA levels in sedentary young men [45]. Hence, there are controversies in the results of studies evaluating the effects of CoQ10 supplementation on inflammation and oxidative stress in various diseases. Possible explanations for these controversies may be different health conditions of the subjects, initial levels of plasma CoQ10, initial concentrations of oxidative stress and inflammatory markers, duration, and sample size of the study.

Patients with burn injury usually experience a reduction in serum albumin levels. In fact, high vascular permeability, especially in burned tissue, leads to exudation and transcapillary albumin loss. Additionally, albumin hepatic synthesis decreases in an acute phase response after burn injury [46]. Given the role of albumin in maintaining oncotic pressure, hypoalbuminemia can cause edema in critically ill patients which consequently leads to other complications such as respiratory problems due to pulmonary edema, delayed burn wound healing due to soft tissue edema and gut malabsorption, and diarrhea due to intestinal edema [47]. In a recent prospective cohort study, it was observed that hypoalbuminemia in burn patients was strongly and positively associated with renal failure, pulmonary infection, sepsis, and death and was known as a good predictor [48]. In our investigation, CoQ10 supplementation significantly improved albumin levels in burn patients. Increased vascular permeability following burn injury occurs because of different signaling pathways. After burn injury, adherence of neutrophils to the vascular endothelial cells and a series of changes cause vascular endothelial damage [49]. Studies have reported that CoQ10 can prevent the infiltration of neutrophils and reduce endothelial barrier dysfunction [50]. Moreover, various inflammatory mediators and oxidants are involved in endothelial barrier dysfunction after burn [49]. The antioxidant and anti-inflammatory properties of CoQ10 can help improve vascular integrity and reduce albumin loss [50]. Although in the present clinical trial, supplementation with CoQ10 could not reduce evaluated inflammatory factors and oxidative stress markers in burn patients, it should be noted that other inflammatory factors and oxidative stress markers were not measured in this study. However, due to a lack of compelling evidence, future clinical trials, powered for albumin as the primary outcome, must be designed to confirm or refute this effect.

We also evaluated the effect of CoQ10 intake on some clinical outcomes. CoQ10 supplementation could not significantly affect blood pressure, body temperature, O_2_ saturation, ICU length of stay, and 28-mortality rate compared to placebo in burn patients. In their study, Donnino et al. observed no significant difference in ICU length of stay and in-hospital mortality in patients with severe sepsis or septic shock receiving 200 mg ubiquinol twice a day for one week compared to placebo [42]. In another study on septic patients, administration of 100 mg CoQ10 twice a day for 7 days significantly reduced in-hospital mortality but had no effect on ICU length of a stay [28]. Hasanloei et al. conducted a study investigating the effect of 400 mg/day sublingual CoQ10 for 7 days on patients with traumatic injury admitted to ICU compared to placebo. They observed that CoQ10 administration considerably reduced ICU and hospital length of stay and mechanical ventilation duration [39]. Future studies on critically ill patients, especially burn patients, are needed to determine the impact of CoQ10 supplementation on clinical outcomes in these patients.

Our study had a number of limitations. First, the sample size of the study was relatively small, and the duration of the intervention was almost short. Because the possibility of patient loss due to death or discharge, it was not possible to increase the duration of the intervention to more than 10 days. Second, due to limited funding, we could not evaluate more related outcomes especially other oxidative stress and inflammatory markers as well as plasma CoQ10 levels. Moreover, a lack of data about the dosage of drugs received by included patients may affect the results.

## Conclusion

In conclusion, we have shown that CoQ10 supplementation had no significant effect on oxidative stress, inflammatory markers, or metabolic and clinical outcomes of burn patients; however, positive changes in serum albumin levels were observed at the end of the study which needs to be confirmed in future clinical trials. More well-designed clinical trials on burn patients should be done to determine the exact effect of CoQ10 supplementation on the health outcomes of these patients.

## Data Availability

The data supporting this study’s findings are available from the corresponding author upon reasonable request.
